# Bacterial DNA translocation contributes to systemic inflammation and to minor changes in the clinical outcome of liver transplantation

**DOI:** 10.1038/s41598-018-36904-0

**Published:** 2019-01-29

**Authors:** Gonzalo P. Rodríguez-Laiz, Pedro Zapater, Paola Melgar, Cándido Alcázar, Mariano Franco, Paula Giménez, Sonia Pascual, Pablo Bellot, José M. Palazón, María Rodríguez, Fernando Carnicer, Patricio Más-Serrano, José M. González-Navajas, Luís Gómez, José Such, Félix Lluís, Rubén Francés, on behalf of the Liver Transplantation Group, Carlos de Santiago, José Navarro, Francisco Martínez, María Galiana, Esteban Salas, Inmaculada Palomar, Javier Irurzun, Juan Matías Bernabé, Miguel Perdiguero, María Díaz, Teresa Lozano, Esperanza Merino, Susana Almanza, José M Mataix, Pedro Orts, Francisco Jaime

**Affiliations:** 1 Servicio de Cirugía General y del Aparato Digestivo, HGUA, Alicante, Spain; 2 Instituto de Investigación Sanitaria y Biomédica de Alicante (ISABIAL-Fundación FISABIO), Alicante, Spain; 3 Servicio de Farmacología Clínica, Hospital General Universitario, Alicante, Spain; 4 CIBERehd, Instituto de Salud Carlos III, Madrid, Spain; 5 Unidad Hepática, Hospital General Universitario, Alicante, Spain; 6 Servicio de Farmacia, Hospital General Universitario, Alicante, Spain; 7 Servicio de Anestesiología, Reanimación y Terapia del Dolor, Hospital General Universitario, Alicante, Spain; 8 Digestive Disease Institute, Cleveland Clinic Abu Dhabi, Abu Dhabi, UAE; 9 Case Western Reserve University, Ohio, OH USA; 10 Dpto. Medicina Clínica, Universidad Miguel Hernández, San Juan, Spain; 11 Coordinación de trasplantes, Hospital General Universitario, Alicante, Spain; 12 Servicio de Radiología, Hospital General Universitario, Alicante, Spain; 13 Servicio de Nefrología, Hospital General Universitario, Alicante, Spain; 14 Servicio de Cardiología, Hospital General Universitario, Alicante, Spain; 15 Servicio de Medicina Interna, Hospital General Universitario, Alicante, Spain; 16 Unidad de Cuidados Intensivos, Hospital General Universitario, Alicante, Spain

## Abstract

Bacterial (bact)DNA is an immunogenic product that frequently translocates into the blood in cirrhosis. We evaluated bactDNA clearance in patients undergoing liver transplantation (LT) and its association with inflammation and clinically relevant complications. We prospectively included patients consecutively admitted for LT in a one-year follow-up study. We evaluated bactDNA before and during the first month after LT, quantifying cytokine response at 30 days. One hundred patients were included. BactDNA was present in the blood of twenty-six patients undergoing LT. Twenty-four of these showed bactDNA in the portal vein, matching peripheral blood-identified bactDNA in 18 cases. Thirty-four patients showed bactDNA in blood during the first month after LT. Median TNF-α and IL-6 levels one month after LT were significantly increased in patients with *versus* without bactDNA. Serum TNF-α at baseline was an independent risk factor for bactDNA translocation during the first month after LT in the multivariate analysis (Odds ratio (OR) 1.14 [1.04 to 1.29], P* = *0.015). One-year readmission was independently associated with the presence of bactDNA during the first month after LT (Hazard ratio (HR) 2.75 [1.39 to 5.45], P = 0.004). The presence of bactDNA in the blood of LT recipients was not shown to have any impact on complications such as death, graft rejection, bacterial or CMV infections. The rate of bactDNA translocation persists during the first month after LT and contributes to sustained inflammation. This is associated with an increased rate of readmissions in the one-year clinical outcome after LT.

## Introduction

Cirrhosis is an end-stage liver disease reached after long-term progression of fibrosis and sustained inflammation^1^. Secondary to chronic liver failure, patient progress is frequently hampered by clinically relevant complications, some of which can be directly related to the persistent inflammatory environment^2–4^.

The immunological dysfunction observed in cirrhosis^5^, along with increased gut barrier permeability and microbiota dysbiosis, contributes to a higher translocation rate of t immunogenic antigens from the gut into the circulatory system of patients^6^. It is important to stress that these transient episodes are not related to the development of overt infections. The translocation of gut bacterial products into the blood has been extensively documented in cirrhotic patients with non-infected ascitic fluid^7,8^. However, these episodes are in fact related to an exacerbation of the inflammatory response^9–11^, systemic circulatory abnormalities and intrahepatic endothelial dysfunction^12^, or even an increased mortality rate at one year among patients with decompensated cirrhosis^13^.

We previously observed in another setting how these episodes were cleared after bariatric surgery^14^ in nearly all morbidly obese patients, who also show a sustained inflammatory response and gut-derived antigenic translocation into the circulatory system (without overt infections). This suggests that a severe reduction in the inflammatory burden and antigenic translocation could help improve patient progress. In fact, the HOMA-2 index remains significantly increased after bariatric surgery despite massive weight loss in those patients who do not clear antigens in blood^14^.

Based on the above, the main purpose of our study was to discover whether liver transplantation in patients with advanced cirrhosis might also reduce the translocation of antigenic products into the circulatory system of patients and the associated systemic inflammatory response. Furthermore, due to the fact that we have previously demonstrated the association between the presence of these antigens in blood and clinical complications in patients with cirrhosis, we followed up all LT recipients for a year after surgery.

## Patients and Methods

### Patients and study design

We conducted a prospective study in cirrhotic patients consecutively admitted for LT to the Hospital General Universitario de Alicante (HGUA) between 2012 and 2015. All patients were listed following the implementation of a sickest-first prioritisation system using the Model for End-Stage Liver Disease (MELD) score, with hepatocellular carcinoma (HCC) scoring. The listed patients were called for surgery whenever a suitable donor became available. Donors’ information included age, sex, cold ischemia time (defined as the time from donor cross-clamping to the removal of the organ from the cold preservation solution), warm ischemia time (defined as the time from the end of the cold ischemia to graft reperfusion), and donor risk index. Contraindications for liver transplantation were in accordance with international guidelines^15^. No organs were procured from prisoners. All organs were obtained through the National Transplant Organization of Spain (www.ont.es/paginas/home.aspx), the only certified government agency responsible for allocating, distributing and auditing transplants in the country. Patients receiving multiple organ transplants or retransplants were excluded from the study.

**Figure 1:**
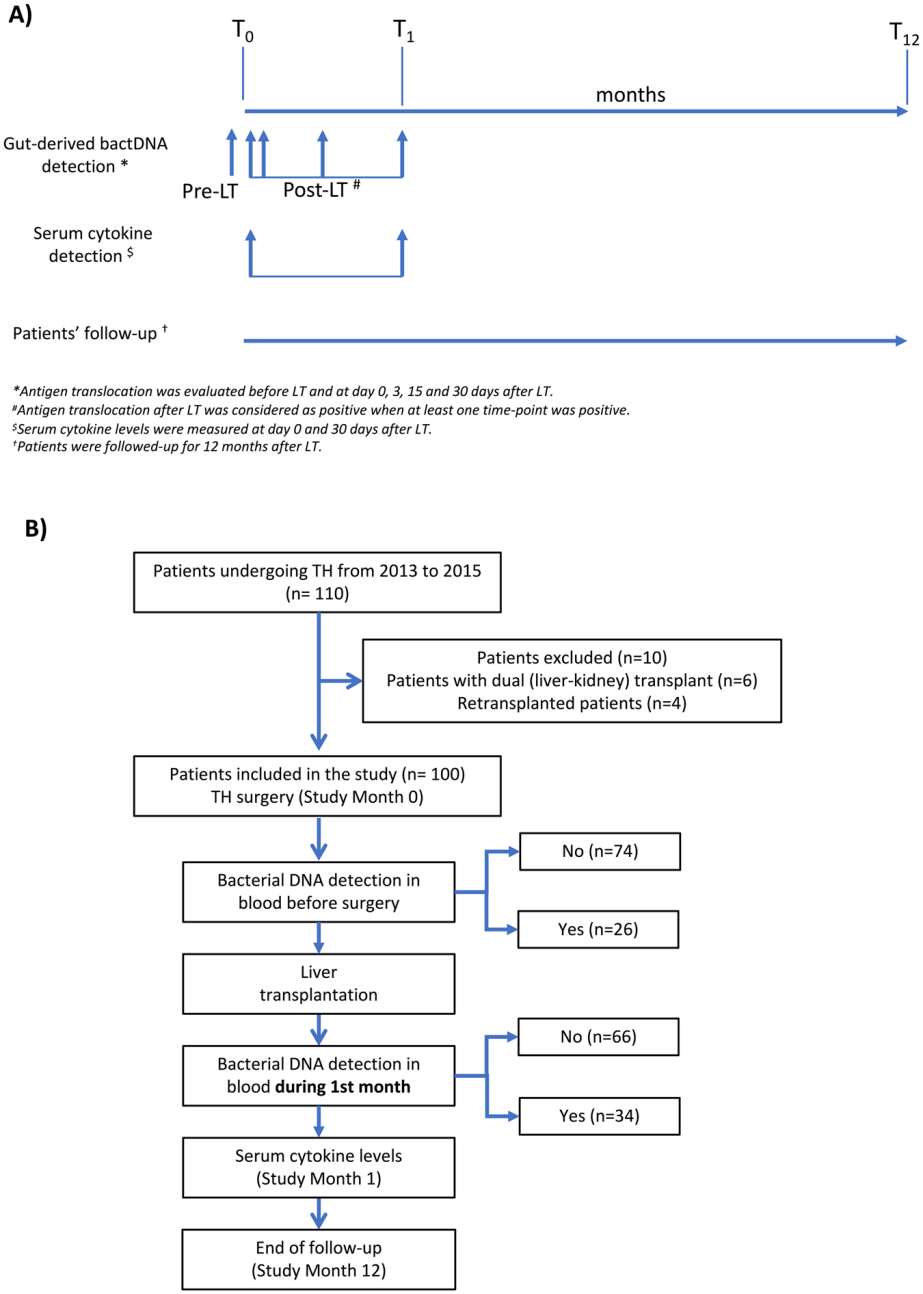
Study design (**A**) and patient inclusion (**B**).

The study design is presented in Figure 1A. Peripheral blood samples were obtained from donors, and LT recipients at surgery, and 3, 15 and 30 days after surgery for routine haematological and biochemical studies, and inoculated in individual 10 ml aerobic and anaerobic blood culture bottles. Two separate peripheral blood samples, and one from recipients’ portal vein at the time of surgery were simultaneously inoculated under aseptic conditions in rubber-sealed sterile Vacutainer SST II and K3E tubes, respectively (BD Diagnostics, Erembodegem, Belgium) that were not exposed to the air at any time.

All included patients gave informed consent to participate in the study and the Ethics Committee of HGUA approved the study protocol. All methods were performed in accordance with relevant guidelines and regulations.

### Surgical technique

Fluid restriction was implemented throughout the operation. All but one of the procedures were performed with inferior vena cava preservation and with a temporary porto-caval shunt. All biliary reconstructions were performed duct-to-duct without T-tube. In an attempt to minimise biliary complications, either arterial (9 cases) or simultaneous arterial and portal (29 cases) reperfusion was performed in patients whose donors were aged ≥ 70 years or in donors after cardiac death. Abdominal drains were used in the first 19 cases. Thromboelastometry (ROTEM®) was used throughout the operating time as a guide to correct coagulopathy and help minimise blood loss and blood replacement. Early extubation was defined according to Mandell^16^ as removal of the endotracheal tube immediately following surgery. Oral intake was liberally commenced a few hours later, and an abdominal Doppler ultrasonography was performed within the first 12 hours. Patient ambulation started soon after, often on the day of transplant or the first day after transplantation.

### Immunosuppression

Standard immunosuppression was achieved with a regime of steroids, once-daily tacrolimus, and mycophenolate mofetil. Patients with renal dysfunction received basiliximab induction, followed by delayed initiation of tacrolimus. An intensive pharmacokinetic monitoring programme was implemented from day one of treatment. Target levels were chosen for each patient according to kidney function, cirrhosis etiology (e.g. HCV, alcohol) and biochemical liver function tests after transplantation.

An extensive description of surgical procedure and immunosuppression is available as supplementary material (Suppl. Materials and Methods).

### Patient follow-up

Adverse outcomes were recorded and graded using the classification by Dindo *et al*.^17^ into five major grades (I-V) including 2 subgroups (IIIa,b and IVa,b) with death graded the highest (V). Patients were initially followed up twice a week on an outpatient basis and simultaneously attended by a hepatologist, a surgeon and a pharmacist. Abdominal CT scans were initially obtained every three months for patients who received a transplant for HCC. Hospital readmissions due to scheduled procedures not related to clinical or surgical complications were not considered in the follow-up analysis. Data was collected for this study until one year after the LT procedure.

### Quantification of bactDNA fragments, serum endotoxin and cytokine levels

Genomic DNA was isolated from 5 × 10^6^ cells using the QIAamp DNA Blood Minikit (Qiagen, Hilden, Germany). BactDNA was identified by running a broad-range PCR with 5′-AGAGTTTGATCATGGCTCAG-3′ as forward and 5′-ACCGCGACTGCTGCTGGCAC-3′ as reverse universal eubacterial primers of a conserved region of the 16SrRNA gene, followed by partial nucleotide sequencing. Full methodology description including specificity and sensitivity are described elsewhere^9^. Patients above the threshold for bactDNA detection of 10 pg were considered as bactDNA-positive. PCR amplicons were loaded onto DNA Lab-on-a-chip (Agilent Technologies, Palo Alto, CA) and analysed with an Agilent 2100 BioAnalyzer. This was followed by a quantitative chromogenic limulus amebocyte lysate (LAL) test (BioWhittaker, Nottingham, UK) to evaluate endotoxin levels in blood and AF samples as previously described^18^.

Serum TNF-α and IL-6 levels were determined using the Cytometric Bead Arrays (CBA) flow cytometry in a FACSCanto II (Becton Dickinson, San Jose, CA) according to the manufacturers’ instructions. The detection limit for each cytokine assay ranged from 2–5 pg/mL.

### Statistical analysis

Categorical variables are reported as frequency or percentages. Descriptive statistics for normally and non-normally distributed continuous data are reported as mean ± standard deviation (SD) or median (lower quartile, upper quartile), respectively. The normality of the distribution of continuous variables was assessed using the Shapiro–Wilk test, with the aid of a visual inspection. Categorical variables were compared with the Chi-square test or Fisher exact test, when appropriate. Differences between groups for normally and non-normally distributed quantitative data were analysed using the independent samples t-test or the Mann-Whitney U test, respectively. Statistical differences between three or more groups were analysed using the ANOVA test with Bonferroni correction for multiple comparisons in normally distributed data or the Kruskal–Wallis test with post-hoc pairwise comparisons using Mann-Whitney U test and Bonferroni correction in non-normally distributed data.

Variables listed in Table 1 were analysed as possible predictors of the presence of bactDNA at baseline and during the month after LT using logistic regression analysis. Variables attaining statistical significance (P ≤ 0.05) in previous univariate analysis were entered into a multiple logistic-regression analysis using a forward stepwise method. Logistic regression results are reported as odds ratio (OR) and 95% confidence interval (95% CI).

**Table 1:** Clinical and analytical characteristics of patients.

Variable	All patients (n = 100)	Without bacterial DNA before LT (n = 75)	With bacterial DNA before LT (n = 25)	*p*
	Mean ± SD or N (%)	Mean ± SD or N (%)	Mean ± SD or N (%)	
Age (years)	57.4 ± 8.1	56.9 ± 8.4	58.4 ± 7.3	0.59
Sex (male/female)	83/17	62/13	21/4	0.89
MELD score	16.4 ± 6.2	16.2 ± 5.8	17.2 ± 7.4	0.47
Lab MELD Score	15.8 ± 7.2	15.7 ± 7.6	16.0 ± 6.0	0.45
Etiology (n)				
Alcohol	44 (44%)	36 (48%)	8 (32%)	0.058
HCV	21 (21%)	16 (21.3%)	5 (20%)	
Alcohol + HCV	19 (19%)	13 (17.3%)	6 (24%)	
Other	16 (16%)	10 (13.3%)	6 (24%)	
HCC (n)	51 (51%)	37 (49.4%)	13 (52%)	0.91
Previous refractory ascites (n)	19 (19%)	15 (20%)	4 (12%)	0.77
Previous SBP episodes (n)	10 (10%)	9 (12%)	1 (4%)	0.45
Previous variceal bleeding (n)	23 (23%)	20 (26.7%)	6 (24%)	0.99
TIPS (n)	3 (3%)	1 (1.4%)	2 (8%)	0.16
Previous hepatic encephalopathy (n)	36 (36%)	28 (37.4%)	8 (32%)	0.68
SID (n)	26 (26%)	24 (32%)	2 (8%)	0.017
Anti-HCV (n)	7 (7%)	5 (6.6%)	2 (8%)	0.99
Use of beta-blockers (n)	45 (45%)	34 (45.4%)	11 (44%)	0.93
Use of antibiotics (n)	2 (2%)	1 (1.4%)	1 (4%)	0.45
Use of PPIs (n)	40 (40%)	28 (37.4%)	12 (48%)	0.61
Mean arterial pressure (mmHg)	82 ± 10	83 ± 10	82 ± 10	0.87
Heart rate (beats/minute)	79 ± 13	80 ± 9	78 ± 12	0.78
Temperature (°C)	36.5 ± 0.5	36.5 ± 0.5	36.5 ± 0.5	0.99
Bilirrubin (mg/dL)	4.7 ± 7.8	4.2 ± 6.8	4.8 ± 6,3	0.75
Albumin (g/dL)	3.4 ± 2.5	3.4 ± 2.2	3.0 ± 2.4	0.45
Quick (%)	60.6 ± 19.8	61.5 ± 15.7	59.0 ± 14.2	0.64
INR	1.8 ± 1.7	1.7 ± 1.5	1.8 ± 1.6	0.78
Serum creatinine (mg/dL)	1.1 ± 0.7	1.0 ± 0.6	1.1 ± 0.6	0.88
Serum Sodium (mEq/L)	136.1 ± 4.3	137.5 ± 3.8	136.4 ± 4.0	0.79
Serum potassium (mEq/L)	4.2 ± 0.6	4.1 ± 0.5	4.3 ± 0.5	0.54
Platelets/mm^3^	108.58 ± 68.60	120.62 ± 46.50	104.20 ± 60.50	0.45
Blood WBC/mm^3^	6444 ± 4012	6679 ± 3894	6210 ± 3850	0.66

SID: selective intestinal decontamination; SBP: spontaneous bacterial peritonitis; TIPS: transjugular intrahepatic portosystemic shunt; PPIs: proton-pump inhibitors; WBCs: white blood cells.

Logistic regression was also used to analyse possible predictors of the most relevant clinical complications observed during the year after LT. Variables listed in Table 1, adverse outcomes, and detection of bactDNA in the first month after LT were included in the analysis.

The Kaplan-Meier life-table was used to analyse timeframe differences between LT and the most relevant clinical complications due to the fact that precise follow-up termination or event dates were known. Data were censored at 365 days. The Log-rank test was used to compare survival curves. Variables with a P value of ≤ 0.05 in previous univariate analysis were entered into multiple Cox proportional-hazards regression and hazard ratios, with the calculation of a 95% CI for event-free overall survival.

All reported P values are two-sided, and P values lower than 0.05 are considered to be significant. All calculations were performed using the R-2.14.1 2011 software (The R Foundation for Statistical Computing).

## Results

### Patient characteristics

One hundred and ten consecutive patients undergoing LT were initially considered in the study. Six dual liver-kidney transplant patients and four retransplant patients were excluded (Fig. 1B). All of the liver explants were obtained from brain dead donors with a mean age of 61 ± 18 years. The median donor risk index was 1.9 (P_25-75_: 1–2). Table 1 details the clinical and analytical characteristics of patients at transplantation. The mean age of patients was 57.4 ± 8.1 years and 83 were male. The mean MELD score at the time of transplant was 16.4 ± 6.2. The main etiology for cirrhosis was alcohol and 51 patients (51%) had HCC.

Mean cold ischemia time was 305 ± 93 min. Mean warm ischemia time was 42 ± 7 min. Mean operating time was 323 ± 66 min. Fifteen patients (15%) received a mean of 2.2 ± 0.7 units of heterologous packed red blood cells from the blood bank in the operating room. A median 500 mL (P_25-75_: 325–745) was recovered in the 45 patients without HCC using a cell saver. All patients were extubated upon completion of the procedure except for one, whose graft never worked due to primary graft non-function. The median stay in the ICU was 15 hours (P_25-75_: 10–29). No patient had to be transferred back to the ICU following their discharge to the surgical ward. The median hospital stay after transplantation was 4 days (P_25-75_: 3–6).

### Dynamics of bactDNA translocation

Prior to LT, bactDNA was present in the peripheral blood of 26 patients and in the portal vein blood of 24 patients. Following surgery, circulating bactDNA was shown in the peripheral blood of 34 patients (34%) in at least one out of three measurements during the first month after LT (Supplementary Table 1). BactDNA had been shown in the peripheral blood of 23 of these patients before LT. Concentration of amplified bactDNA in blood was 29.4 ± 8.6 ng/μL before LT and 31.4 ± 8.22 ng/μL during the first month after surgery (P = 0.58).

All sequenced bacterial genomic fragments are identified in Supplementary Table 2A. Identified species in patients with bactDNA in peripheral and portal blood matched in all cases. Identified species in patients with bactDNA before surgery and after LT also matched in all cases. Supplementary Figure 1 shows the relationship between microbiologic culture and sequencing analysis identification of bacterial species in patients. Overall, species were identified in 35.8% of cases using both microbiologic culture and molecular tools. In addition, 42.8% were identified using molecular tools only and 10.7% with microbiological culture only. The remaining 10.7% cases showed mismatched identifications by both techniques.

Clinical characteristics of patients distributed according to bactDNA status before surgery can be followed in Table 1. No significant clinical or analytical differences were observed, except for patients who were on selective intestinal decontamination (SID).

BactDNA was identified in peripheral blood samples from eight donors (8%), out of which, two LT recipients showed bactDNA before surgery and four showed bactDNA one month after surgery. Bacterial species identification can be followed in Supplementary Table 2B in each case. The persistence of the same bacteria-derived genomic fragments in donor and in recipient was found in one single case (*E. coli*).

### Serum endotoxin levels in LT recipients at baseline

Serum endotoxin levels in LT recipients at baseline (0.68 ± 0.42 UE/mL) were significantly increased in patients with *versus* without bactDNA at baseline (0.98 ± 0.41 *versus* 0.57 ± 0.34 UE/mL; P = 0.001). However, no correlation was found between serum endotoxin levels and the concentration of amplified bactDNA in patients with bacterial antigen (R = −0.11, P = 0.54).

### Serum cytokine response in LT recipients

**Figure 2:**
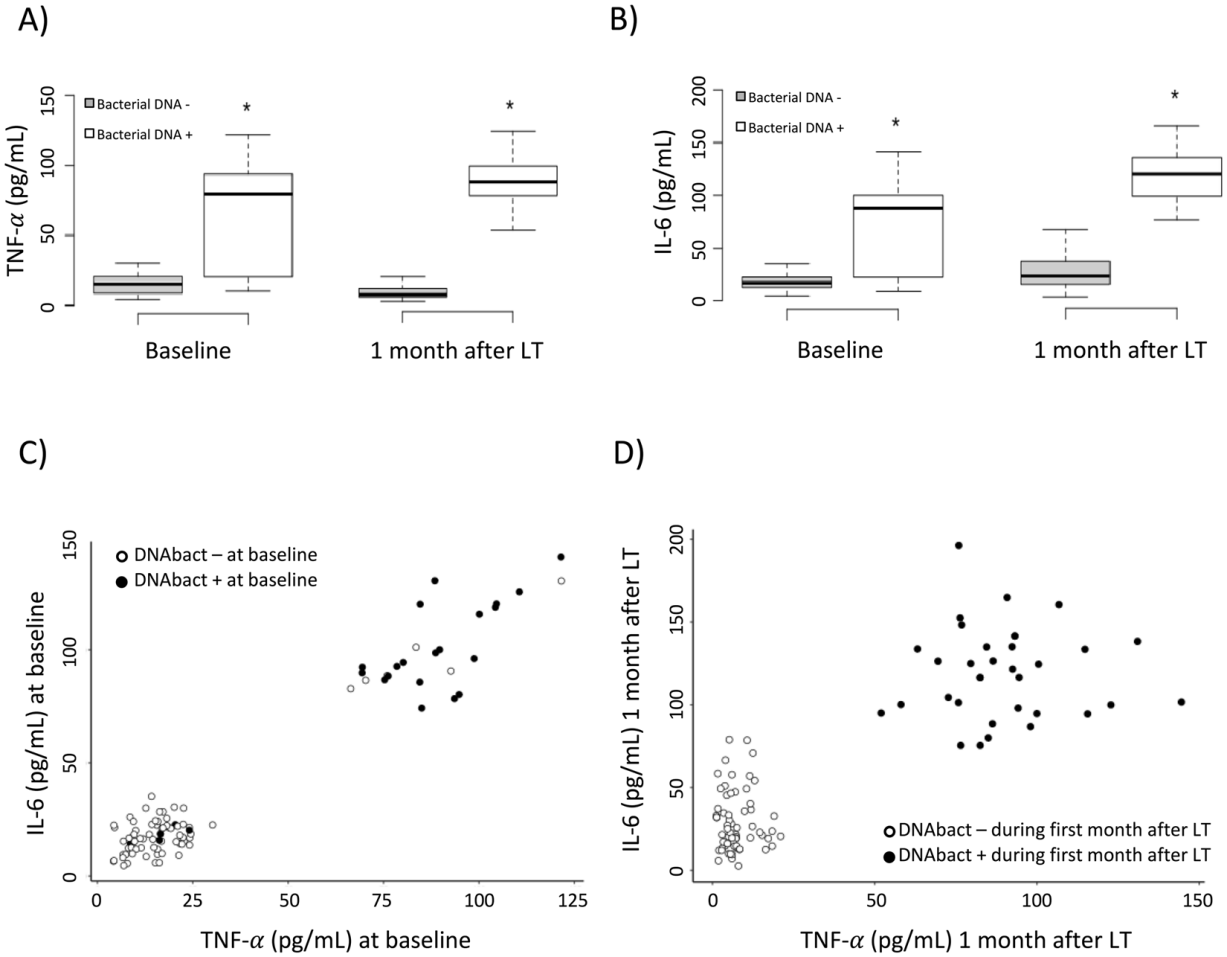
Serum TNF-α (**A**) and IL-6 (**B**) levels in LT recipients before and 30 days after LT and distributed by the presence of bactDNA during the first month after LT. (**C**) correlation between pro-inflammatory cytokines at baseline in LT recipients distributed by the presence of bactDNA at baseline. (**D**) Correlation between pro-inflammatory cytokines one month after LT in patients distributed by the presence of bactDNA at baseline. Median and min/max values are represented. *p < 0.01 compared with patients without bactDNA.

TNF-α and IL-6 were evaluated before and one month after LT in all included patients as shown in Fig. 2. Baseline median TNF-α was significantly increased in patients with *versus* without bactDNA. Similarly, baseline IL-6 serum levels were significantly higher in patients with bactDNA (Fig. 2A). One month after surgery, median TNF-α and IL-6 were also significantly increased in patients with bactDNA during the first month after LT versus those without bactDNA(Fig. 2B).

Serum levels of TNF-α and IL-6 showed a significant correlation in LT patients both at baseline (R = 0.74; P = 0.001, Fig. 2C) and 1 month after LT (R = 0.66; P = 0.001, Fig. 2D). Interestingly, two cytokine-related populations are clearly identified in terms of bactDNA presence. There was a significant correlation between the concentration of amplified bactDNA at baseline and serum levels of TNF-α (R = 0.64; P = 0.001) or IL-6 (R = 0.54; P = 0.001) at baseline. Significant correlations were also observed between endotoxin levels and TNF-α (R = 0.7; P* < *0.001) or IL-6 (R = 0.67; P* < *0.001) at baseline.

Serum TNF-α and IL-6 levels, as well as endotoxin at baseline, were the only variables significantly related to the presence of bactDNA at baseline in the univariate analysis. Serum endotoxin levels were the only to remain statistically significant in the multivariate analysis (Table 2). On the other hand, the absence of HCC, the presence of refractory ascites, the use of beta-blockers, the presence of bactDNA at baseline, serum TNF-α, IL-6 and endotoxin levels before surgery, were significantly associated with the presence of bactDNA in the univariate analysis during the first month after surgery. Only baseline serum TNF-α and endotoxin levels remained as an independent risk factor of bactDNA translocation during the first month after LT in the multivariate analysis (Table 3).

**Table 2:** Variables significantly related to the presence of bacterial DNA at baseline.

	Patients with bactDNA at baseline	(pg/mL)	OR (95%CI)	*P value*
***Univariate Analysis***				
TNF-alpha at baseline	no	20.23 ± 20.94	1.05 (1.04 to 1.07)	0.009
	yes	73.26 ± 33.35		
IL-6 at baseline	no	23.55 ± 23.31	1.05 (1.03 to 1.06)	0.008
	yes	82.44 ± 39.20		
LPS at baseline	no	0.50 ± 0.28	324.0 (46.12 to 4329.0)	0.001
	yes	1.19 ± 0.32		
***Multivariate Analysis***				
			OR (95%CI)	*P value*
TNF-alpha at baseline			1.05 (0.98 to 1.13)	0.18
IL-6 at baseline			1.01 (0.94 to 1.07)	0.95
Endotoxin at baseline			61.2 (7.6 to 1065.0)	0.001

**Table 3:** Variables significantly related to the presence of bacterial DNA during the first month after LT.

	Patients with bactDNA during the first month	N (%) or (pg/mL)	OR (95%CI)	*P value*
***Univariate Analysis***				
HCC (yes)	no	44/66 (66.6%)	0.27 (1.11 to 0.64)	0.003
	yes	12/34 (35.3%)		
Refractory ascites at baseline (yes)	no	8/66 (12.1%)	3.02 (1.07 to 8.83)	0.038
	yes	10/34 (29.4%)		
Use of B-blockers at baseline (yes)	no	27/66 (40.9%)	2.33 (1.01 to 5.56)	0.050
	yes	21/34 (61.7%)		
BactDNA at baseline (yes)	no	7/66 (10.6%)	10,676 (3.95 to 31.94)	0.001
	yes	19/34 (55.8%)		
TNF-alpha at baseline	no	17.18 ± 14.39	1.06 (1.04 to 1.09)	0.001
	yes	66.71 ± 37.09		
IL-6 at baseline	no	20.79 ± 18.40	1.05 (1.03 to 1.07)	0.008
	yes	73.93 ± 42.44		
Endotoxin at baseline	no	0.51 ± 0.31	27.6 (7.7 to 126.5)	0.001
	yes	1.00 ± 0.42		
***Multivariate Analysis***				
			OR (95%CI)	*P value*
HCC (yes)			0.25 (0.004 to 0.21)	0.098
Refractory ascites (yes)			3.03 (0.47 to 19.09)	0.23
B-blockers (yes)			0.92 (0.20 to 3.86)	0.91
BactDNA at baseline (yes)			1.91 (0.13 to 19.55)	0.59
TNF-alpha at baseline			1.14 (1.04 to 1.29)	0.015
IL-6 at baseline			0.94 (0.86 to 1.02)	0.14
Endotoxin at baseline			2.58 (0.32 to26.7)	0.39

### Clinical evolution of LT recipients

Eighty-eight out of the 100 patients developed some complication during the 12-month follow-up period, 63 of them during the first month after LT. Supplementary Table 3 summarises the complete list of complications occurring in these timeframes. Bacterial infectious complications (n = 27), CMV infection (n = 28), biliary/pancreatic complications (n = 18), intraabdominal collection/hemoperitoneum (n = 15), and graft rejection (n = 12) were the ones that most frequently occurred overall. Sixteen patients (16%) died during the 12-month follow-up period, 10 of whom died during the first month after LT. Causes of death were multiorgan failure (n = 8), graft dysfunction (n = 4), infectious complications (n = 2); HCC/neoplasia (n = 1), and biliary/pancreatic complications (n = 1).

**Figure 3:**
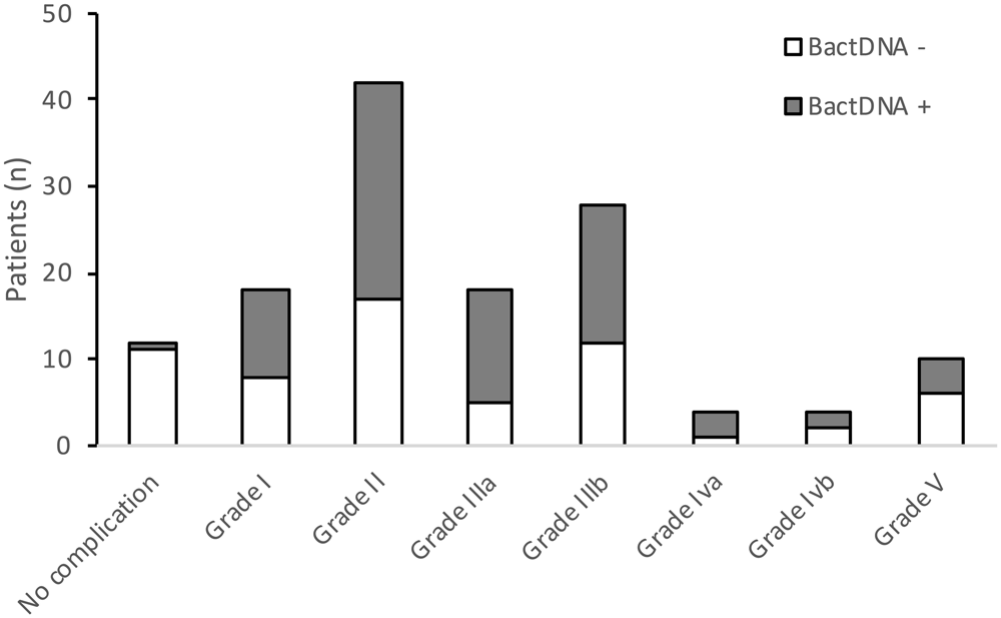
Patients with or without bactDNA during the first month after LT developing complications during the 12-month follow-up according to the *Dindo et al*. classification.

Figure 3 shows the number of patients with and without bactDNA during the first month after LT who presented any grade of complication according to Dindo *et al*. (17).We can see that 11 patients without bactDNA and 1 patient with bactDNA did not show any complication during the 12-month follow-up period (17% *versus* 3%; P = 0.02). Conversely, Grade II (P = 0.02) and Grade IIIa (P = 0.01) complications were significantly more frequent in patients with *versus* without bactDNA.

The association between all clinical and analytical variables and the risk of developing complications (yes/no) at 30 days was evaluated (Table 4). The multivariate analysis showed that the use of proton pump inhibitors (PPIs) at baseline (OR 2.95 [1.12 to 8.24], P = 0.032) and the days hospitalised (OR 1.29 [1.07 to 1.65], P = 0.018) were independently associated with the occurrence of complications during the first month after LT. On the other hand, systemic inflammation and the presence of bactDNA in blood during the first month after LT were significantly associated in the univariate analysis with the risk of developing clinical complications (yes/no) at 1 year after LT. However, none of these variables were independently associated in the multivariate analysis with an increased risk of developing complications during the first year after LT (Supplementary Table 4). None of the clinical or analytical parameters evaluated were associated with the number of complications either during the first month or the first year after LT.

**Table 4:** Variables significantly related to developing clinical complications during the first month after LT.

	Patients with complications during the first 30 days after LT	N (%) or (pg/mL)	OR (95%CI)	*P value*
***Univariate Analysis***				
HCC	no	27/37 (73%)	0.32 (0.13 to 0.74)	0.010
	yes	29/63 (46%)		
PPIs at baseline	no	9/37 (24.3%)	2.65 (1.11 to 6.8)	0.034
	yes	29/63 (46%)		
Hospitalisation (days)	no	4.03 ± 2.09	1.31 (1.09 to 1.7)	0.019
	yes	7.80 ± 10.75		
***Multivariate Analysis***				
			OR (95%CI)	*P value*
HCC (yes)			0.44 (0.16 to 1.71)	0.093
PPIs at baseline (yes)			2.95 (1.12 to 8.24)	0.032
Hospitalisation (days)			1.29 (1.07 to 1.65)	0.018

The presence of bactDNA during the first month after LT and the inflammatory cytokine levels at 30 days were not associated with an increased risk of developing the most frequently present complications in our series (CMV infection, bacterial infections, biliary/pancreatic complications, death, intra-abdominal collection or graft rejection) at 1 year after LT (Supplementary Table 4). Sixty-two patients required readmission during the 12-month follow-up period. Readmission during the 12-month follow-up was due to bacterial infectious complications (n = 18); graft rejection (n = 12); biliary/pancreatic complications (n = 9); upper gastrointestinal bleeding (n = 4); fever (n = 4); tacrolimus neurotoxicity (n = 3); evisceration (n = 3); intra-abdominal collection/haemoperitoneum (n = 3); HCC/neoplasia (n = 2); deep venous thrombosis (n = 1); portal thrombosis (n = 1); seizure (n = 1); and atrial fibrillation (n = 1).

Thirty-one patients required readmission within 30 days after LT. Systemic inflammation at baseline, and at 30 days after LT, as well as the presence of bacterial DNA in blood during the first 30 days after LT were significantly associated in the univariate and multivariate analyses with the need for readmission during this period (Supplementary Table 5). Eighteen out of the 31 patients (58%) who were readmitted in the first 30 days after LT showed bacterial DNA compared with 16 out of 69 patients (23%) with bactDNA who were not readmitted (P* = *0.001). In terms of inflammatory cytokines, TNF-α and IL-6 levels in serum at 30 days after LT were significantly increased in patients who were readmitted *versus* those who were not readmitted (TNF-α: 75.9 pg/mL [8.2–88.7] *versus* 7.4 pg/mL [4.7–18.4], P = 0.001; IL-6: 86.8 pg/mL [21.9–124.7] *versus* 33 pg/mL [19.2–75.5], P = 0.03). The multivariate analysis identified bactDNA as the only independent predictor of readmission at 30 days after LT (OR 4.65 [1.86 to 11.35], P = 0.001).

Table 5 shows variables associated with readmission during the first year after LT in the univariate and multivariate analyses. The multivariate analysis identified bactDNA as the only independent predictor of readmission during the first year after LT (OR 7.97 [95%CI 2.53 to 25.22], P = 0.001). Neither the inflammatory-related variables nor the remaining clinical and analytical parameters evaluated were associated with the number of readmissions either during the first month or the first year after LT.

**Table 5:** Variables significantly related to requiring readmission during the first year after LT. Mean and standard deviations are represented.

	Patients with readmissions during the first year after LT	N (%) or (pg/mL)	OR (95%CI)	*P value*
***Univariate Analysis***				
TNF-alpha at baseline	No	19.82 ± 19.23	1.03 (1.01 to 1.05)	0.003
	Yes	42.71 ± 37.96		
IL-6 at baseline	No	22.43 ± 21.54	1.03 (1.01 to 1.05)	0.003
	Yes	48.93 ± 42.68		
BactDNA during the first 30 days after LT (yes)	No	4/38 (10.5%)	7.97 (2.77 to 29.09)	0.001
	Yes	30/62 (48.4%)		
TNF-alpha at 30 days	No	15.69 ± 27.3	1.03 (1.01 to 1.04)	0.001
	Yes	47.59 ± 43.31		
IL-6 at 30 days	No	38.94 ± 31.07	1.02 (1.01 to 1.03)	0.002
	Yes	72.02 ± 52.48		
***Multivariate Analysis***				
			OR (95%CI)	*P value*
TNF-alpha at baseline			0.98 (0.92 to 1.04)	0.54
IL-6 at baseline			1.03 (0.98 to 1.09)	0.29
BactDNA during the first 30 days after LT (yes)			7.97 (2.53 to 25.22)	0.001
TNF-alpha at 30 days			1.01 (0.96 to 1.07)	0.67
IL-6 at 30 days			0.995 (0.98 to 1.02)	0.96

**Figure 4:**
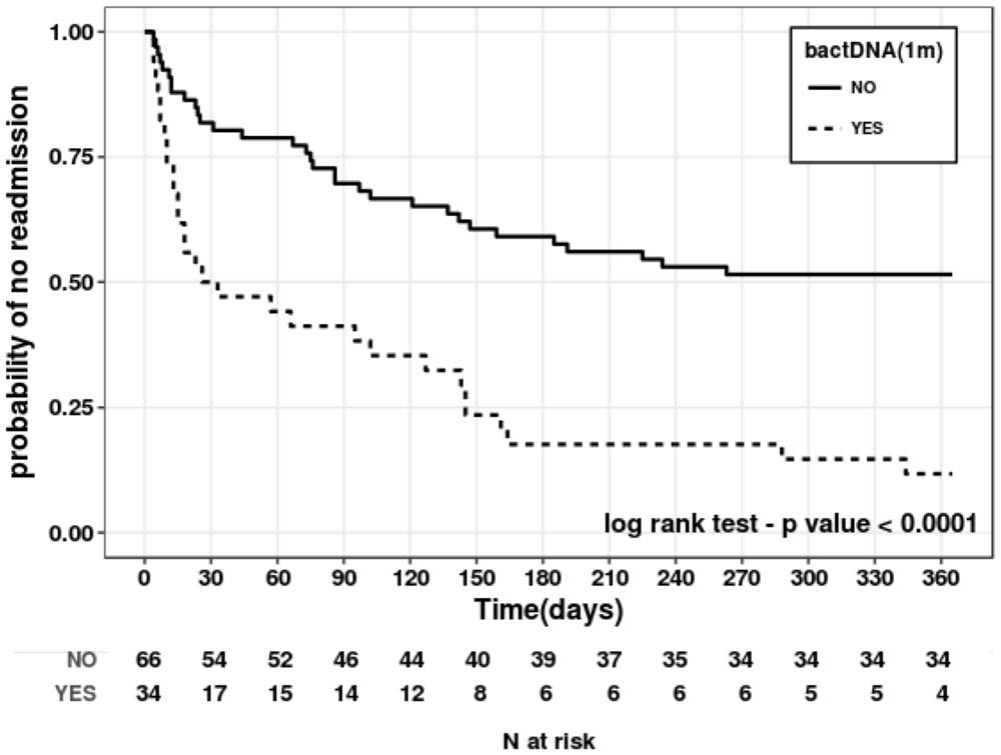
Kaplan-Meier analysis and curve showing the probability of not being readmitted according to the presence of bactDNA during the first month after LT. Log-rank test P value is indicated in the figure.

Figure 4 shows the probability of not being readmitted during one year after LT in patients according to bactDNA in blood during the first 30 days after LT. The Cox proportional hazards multiple regression analysis identified bactDNA as the only independent predictor of no readmission during the first year after LT (HR 2.75 [1.39 to 5.45], P = 0.004). BactDNA presence in blood during the first month after LT was also evaluated for the development of graft rejection and exitus during the 12-month period. However, neither the presence of bactDNA nor the rest of the clinical, analytical or experimental variables showed any association with these complications.

## Discussion

This proof-of-concept study shows that bactDNA translocation rate is not reduced in cirrhotic patients after LT and that it is associated with increased inflammatory cytokine levels. Despite the low impact in the one-year clinical outcome after LT, the sustained inflammatory outlook in LT patients with circulating bactDNA may have long-term consequences that should be tested in new specifically-designed studies with bigger cohorts and longer follow-up periods.

Bacterial translocation (BT) is frequent in patients with cirrhosis. The concept has evolved since its initial definition by *Berg et al*. as the presence of bacteria in cultured mesenteric lymph nodes^19^. BT is now considered to be a physiological event normally kept under control by our immunocompetent cells that becomes pathological in cirrhosis where there is a presence of an immune dysfunction, resulting in its increased frequency^5,6^. But more interestingly, the concept has evolved beyond the translocation of viable bacterial, also considering different bacterial immunogenic products without overt infection^6,20^. The evolution of this concept is a result of years of evidence collected on a significantly increased systemic pro-inflammatory response in a subgroup of non-infected cirrhotic patients reaching levels shown in patients with spontaneous bacterial peritonitis^9,21^. In addition, the translocation of bactDNA has been described as a significant contributor to the development of life-threatening complications in cirrhosis^12,13^. Considering that these patients do not have overt infections, we hypothesise that the presence of bacterial immunogenic products, such as bactDNA, is in fact indirectly reflecting certain unfavourable inflammatory conditions that facilitate the development of complications in patients^13^.

A series of 100 consecutive cirrhotic LT recipients meeting inclusion criteria are included in our study. A recent review of the OPTN/UNOS database identified 66,461 patients who received a primary isolated cadaveric liver transplant over a 20-year period (1993–2012), with a median MELD of 17 at listing, and 20 at transplantation. In our series, MELD was 16 both at listing and at transplantation (unpublished results). These differences can be explained by the higher proportion of HCC among our recipient population and the greater supply of organs in Spain. The results presented in this study lead us to conclude first, that despite cirrhotic liver replacement, the rate of bactDNA translocation remains similar to the rate observed before LT. The observed rate in bactDNA translocation (26%) is similar to the one found by Moharem HA *et al*. in a recent study on hemodynamic and coagulation parameters during living-donor liver transplant (33%)^22^ Also described is the absence of early hepatic clearance of endotoxins after LT^23^. Apart from anatomical changes that are probably induced by sustained portal hypertension over time, two aspects may be relevant in explaining this recurrent antigenic challenge. A leaky gut may temporarily persist due to distorted gut barrier integrity and intestinal bacterial overgrowth typically present in advanced cirrhosis. However established immunosuppressant therapy may also contribute to an increased permissive status. Both aspects may lead to impaired immune function and increase the risk of antigen translocation in these patients. In an attempt to reduce the loss of bactDNA-positive patients due to this antigen transient dynamics into blood^8^, we used a novel approach where we evaluated the presence of bactDNA at up to 4 different time-points during the first month, which probably accounts for the slightly increased rate of bactDNA after LT *versus* before LT. Also worthy of note is the fact that 8% of donors show bactDNA in blood, a similar figure to the one described as physiological translocation in healthy controls^6^ and in line with the detection of endotoxin levels in donors described in the past^24^. It is important to stress that no statistical association was found between bactDNA translocation in the blood of donors and recipients in this study.

Second important result refers to the significantly increased serum levels of TNF-α and IL-6 after LT in patients with bactDNA *versus* without bactDNA translocation. This result mirrors the associated immune response observed in patients with advanced cirrhosis and bactDNA circulating fragments^9,25^, and it further supports the view that LT in cirrhotic recipients does not resolve bactDNA-associated inflammation in this subgroup of patients. This result clearly supports the role of these non-infectious bacterial antigens as a trigger of inflammation. There has been much debate about whether inflammation characteristic of liver damage progression facilitates the translocation of bacterial antigens or if these products increase inflammation and accelerate liver damage^5,6^. Despite their likely interaction with each other, either anatomical or physiological derangements secondary to liver function failure remain present in LT recipients, facilitating bactDNA translocation. These results are in contrast with the significant clearance of bactDNA translocation observed in morbidly obese patients after bariatric surgery^14^.

Finally, based on the fact that bactDNA translocation is considered to be pathological in cirrhosis and that its presence in blood of non-infected patients can be associated with the development of complications at one year^13^, the detection of bactDNA in the blood of LT recipients becomes relevant as a marker of interest for the clinical outcome of patients. In this regard, increased hospital readmission was the only complication from all the ones evaluated during one year after LT that was associated with the detection of bactDNA in LT recipients. Apart from increased health care costs, patients requiring readmission have shown decreased survival compared with those not requiring readmission during the first year after LT^26–28^. Therefore, research needs to be conducted to reduce readmission rates and improve health care costs and survival after LT. Rates and causes, as well as risk factors for readmissions after LT, have been evaluated in the past and vary significantly^26–29^. We found no correlation between the length of hospitalisation after LT and an increased risk of readmission, as shown in other studies^26,30^. More specifically, early discharge from hospital after LT was not associated with an increased risk of readmission, confirming findings from a previous study^31^. Despite this association with increased readmissions, which may be incidental, the presence of bactDNA in the blood of LT recipients was not shown to have any impact on complications such as death, graft rejection, bacterial or CMV infections. Therefore, the specifically tackling of bactDNA translocation does not seem to be a priority in the management of these patients.

In conclusion, our study shows that LT recipients have a similar bactDNA translocation rate and associated soluble inflammatory response to that of advanced cirrhotic patients before surgery. While these episodes are associated with increased readmission rates, they are not associated with any substantially worsened outcomes during the first year after LT.

## Supplementary information

Supplementary Dataset 1

## Data Availability

The authors can provide the materials, data and associated protocols to any reader who wishes to obtain them.
