# Supplementary material for: Bacterial DNA translocation contributes to systemic inflammation and to minor changes in the clinical outcome of liver transplantation
Source: Sci Rep. 2019 Jan 29;9:835. doi: 10.1038/s41598-018-36904-0 (PMC6351615; doi:10.1038/s41598-018-36904-0)

Title:

Bacterial DNA translocation contributes to systemic inflammation and to minor changes in the clinical outcome of liver transplantation

Authors: Gonzalo P. Rodríguez-Laiz[1,2], Pedro Zapater[2,3,4], Paola Melgar[1,2], Cándido Alcázar[1,2], Mariano Franco[1,2], Paula Giménez[2,4], Sonia Pascual[2,4,5], Pablo Bellot[2,4,5], José M. Palazón[2,4,5], María Rodríguez[2,5], Fernando Carnicer[2,5], Patricio Más-Serrano[2,6], José M. González-Navajas[2,4], Luís Gómez[2,7], José Such[8], Félix Lluís[1,2], Rubén Francés[2,4,9], on behalf of the Liver Transplantation Group.

Corresponding author: Rubén Francés, PhD. CIBERehd-HGUA. C/ Pintor Baeza 12, 03010 Alicante, Spain. Tel: +34965913928; Fax: +34965913922; E-mail: frances_rub@gva.es

Supplementary material for Patients and Methods.

Surgical technique

General anesthesia was induced with propofol (1.5-2.0 mg/kg), rocuronium (1 mg/kg) and fentanyl (5 µg/kg), and maintained with sevoflurane. Two indwelling catheters were placed in left radial and right femoral arteries. Two high-flow catheters in left basilic vein and right jugular vein were routinely used, and a pulmonary artery catheter was placed on demand. To prevent surgical site infection, amoxicillin-clavulanate 2g IV was used as perioperative antibiotic prophylaxis. Pressure and cardiac outputs were measured using continuous thermo dilution. Fluid restriction was implemented throughout the surgical procedure. Plasmalyte® and 20% albumin was given at a rate of 4 ml/kg-h to cover for unaccounted fluid losses, and red blood transfusion was given for hemoglobin <7 g/dl, and/or venous oxygen saturation <70%.

Routine preoperative hemoextraction (whole blood) was performed when preoperative hemoglobin ≥9 g/dl. The maximum amount drawn was 3 units, and extraction was stopped when venous oxygen saturation <70%. The blood was stored at room temperature in the operating room and reinfusion was started on demand during the biliary reconstruction. A cell-saver was used in patients who didn't have a diagnosis of malignancy or any intraoperative evidence of contamination or infection.

All procedures were performed with inferior vena cava preservation and temporary porto-caval shunt (TPCS). Grafts were washed prior to reperfusion with 500 ml of Plasmalyte® infused through the portal vein at room temperature. Blood flow was measured in the native portal vein, in the TPCS at completion and prior to its ligation, and in the graft portal vein and hepatic artery at completion and just prior to abdominal closure. All biliary reconstructions were performed duct-to-duct without T-tube. In order to minimize the occurrence of biliary complications, either arterial or simultaneous arterial and portal reperfusion was performed in patients whose donors were ≥75 years old (27 cases). No abdominal drains were used.

Coagulation disorders present at the start of the procedure were not corrected. Thromboelastometry (ROTEM®) was used throughout the operating time as a guide to correct coagulopathy and help minimize blood loss and blood replacement.

Analgesia was obtained with remifentanyl, and a bolus of morphine (0.1 mg/kg) was given right after reperfusion. Sugammadex was given at the end of the procedure to avoid neuromuscular deficit during the early postoperative period. Early extubation was defined according to Mandell (18) as removal of the endotracheal tube immediately following surgery (up to 6 hours). Standard criteria for extubation were: patients followed verbal commands, positive gag reflex, tidal volumes >6 ml/kg, respiratory rate <20, oxygen saturation >95% while breathing spontaneously (FiO_2 ≤50%), normocarbia judged by end tidal CO_2, reversal of neuromuscular blockade judged by peripheral nerve stimulator and clinical assessment, and core body temperature between 36.5 and 37.5°C.

Following extubation, patients were transferred to the ICU with continuous monitoring. Logistics included the availability of 1:1 nursing to patient ratio, and continuous monitoring. Oral intake was started liberally a few hours later, and abdominal Doppler ultrasonography was performed within the first 12 hours after ICU admission. Before the transfer to the surgical ward, arterial lines and pulmonary artery catheters were removed.

The surgical ward was a 27-bed nursing unit dedicated to patients before and after general and digestive surgery, with a 1:10 nurse-to-patient ratio. Patients were monitored initially by continuous pulse oxymetry, and noninvasive blood pressure measurements. Urine output was measured 3 times daily, and body weight and abdominal perimeter were obtained daily. Patients started early ambulation, often on the day of transplant or on the first day postransplant.

Immunosuppression

Standard immunosuppression was achieved with a regime of: i) steroids: methilprednisolone 500 mg IV given immediately after reestablishing the portal

and arterial inflows during the procedure, followed by a 4-day IV and 8-day oral stepwise dose reduction until day 12th, with prednisone 10 mg PO daily until day 90th, and prednisone 5 mg PO daily until day 180th when they are discontinued; ii) tacrolimus (Advagraf®) 0.1-0.15 mg/kg PO daily; and iii) mycophenolate mofetil (1 g/12 h, oral). Patients with renal dysfunction were given induction with basiliximab (Simulect®) followed by delayed start of tacrolimus. An intensive pharmacokinetic monitoring program was implemented from day one of treatment, drawing peripheral blood samples after the first dose including the measurement of trough level (T_0, before the second dose), as well as T_2 (2 hours post dose) and T_3 (3 hours post dose) and daily trough levels thereafter until discharge, and during regular visits to the out-patient clinic. In addition, T_0, T_2 and T_3 levels were obtained on days 15th and 30th after liver transplantation, at 2, 3 and 6 months, and on demand whenever deemed necessary.

Blood samples were immediately assayed using an auto-analyzer (QMS® technology, Indiko® platform - ThermoFisher®). The dose of tacrolimus to achieve a given target steady plasma level of the immunosuppressant was calculated using a Bayesian estimation methodology based on pharmacokinetic individual parameters. Target levels were chosen for each patient according to kidney function, etiology of cirrhosis (ie, HC virus, alcohol), and biochemical liver function tests post-transplantation.

Supplementary Table 1. Bacterial identifications at different time-points after LT.

LT recipient	ID BASAL	ID DAY3	ID DAY 15	ID DAY 30
1	-	-	-	-
2	-	-	-	-
3	-	-	-	-
4	-	-	-	-
5	-	-	-	-
6	-	-	-	-
7	*E COLI*	*E COLI*	.	*E COLI*
8	*STAPHYLOCOCCCUS SPP*	.	*STAPHYLOCOCCUS AUREUS*	*STAPHYLOCOCCUS AUREUS*
9	-	-	-	-
10	-	-	-	-
11	-	-	-	-
12	-	-	-	-
13	-	-	-	-
14	-	-	-	-
15	-	-	-	-
16	-	-	-	-
17	-	-	-	-
18	-	-	-	-
19	-	-	-	-
20	-	-	-	-
21	*E COLI*	*E COLI*	-	*E COLI*
22	*E COLI*	*E COLI*	*E COLI*	-
23	-	-	-	-
24	-	-	-	-
25	-	-	-	-
26	*KLEBSIELLA PNEUMONIAE*	-	*KLEBSIELLA PNEUMONIAE*	-
27	-	-	-	-
28	-	-	-	-
29	-	-	-	-
30	-	-	-	-
31	-	-	-	-
32	-	*E COLI*	*E COLI*	-
33	-	-	-	-
34	-	-	-	-
35	-	-	-	-
36	-	-	-	-
37	*KLEBSIELLA PNEUMONIAE*	*KLEBSIELLA PNEUMONIAE*	*KLEBSIELLA PNEUMONIAE*	-
38	-	*MORGANELLA MORAGANII*	-	*MORGANELLA MORAGANII*
39	-	-	-	-
40	*STAPHYLOCOCCUS AUREUS*	-	*STREPTOCOCCUS PNEUMONIAE*	-
41	-	-	-	-
42	-	-	-	-
43	-	*STREPTOCOCCUS PNEUMONIAE*	-	-
44	-	-	-	-
45	*E COLI*	*E COLI*	*E COLI*	*E COLI*
46	*ENTEROCOCCUS FAECIUM*	-	-	-

47	*-*	*E COLI*	*E COLI*	*-*
48	*-*	*-*	*-*	*-*
49	*E COLI*	*-*	*E COLI*	*E COLI*
50	*ENTEROBACTER SPP*	*-*	*-*	*-*
51	*-*	*-*	*-*	*-*
52	*E COLI*	*E COLI*	*E COLI*	*-*
53	*PROTEUS VULGARIS*	*E COLI*	*E COLI*	*-*
54	*-*	*-*	*-*	*-*
55	*-*	*-*	*-*	*-*
56	*-*	*-*	*STAPHYLOCOCCUS AUREUS*	*STAPHYLOCOCCUS AUREUS*
57	*-*	*-*	*-*	*-*
58	*S AGALACTIAE*	*YERSINIA PSEUDOTUBERCULOSIS*	*-*	*-*
59	*KLEBSIELLA PNEUMONIAE*	*KLEBSIELLA PNEUMONIAE*	*-*	*KLEBSIELLA PNEUMONIAE*
60	*E COLI*	*-*	*-*	*-*
61	*-*	*-*	*-*	*-*
62	*STAPHYLOCOCCUS AUREUS*	*-*	*-*	*-*
63	*-*	*-*	*E COLI*	*-*
64	*E COLI*	*CAMPYLOBACTER JEJUNI*	*-*	*-*
65	*-*	*-*	*-*	*-*
66	*PROTEUS MIRABILIS*	*-*	*-*	*-*
67	*-*	*-*	*-*	*-*
68	*-*	*-*	*-*	*-*
69	*STAPHYLOCOCCUS AUREUS*	*STAPHYLOCOCCUS SPP*	*STAPHYLOCOCCUS HAEMOLITICUS*	*-*
70	*KLEBSIELLA PNEUMONIAE*	*ENTEROCOCCUS FAECIUM*	*-*	*-*
71	*E COLI*	*E COLI*	*-*	*ENTEROBACTER CLOACAE*
72	*STREPTOCOCCUS PNEUMONIAE*	*-*	*-*	*-*
73	*-*	*-*	*-*	*-*
74	*E COLI*	*-*	*E COLI*	*-*
75	*-*	*STAPHYLOCOCCUS LUGDUNENSIS*	*-*	*-*
76	*-*	*-*	*STREPTOCOCCUS SPP*	*STREPTOCOCCUS SPP*
77	*-*	*-*	*-*	*-*
78	*-*	*KLEBSIELLA PNEUMONIAE*	*KLEBSIELLA PNEUMONIAE*	*KLEBSIELLA PNEUMONIAE*
79	*-*	*-*	*-*	*-*
80	*-*	*-*	*-*	*-*
81	*-*	*-*	*-*	*-*
82	*-*	*-*	*-*	*-*
83	*-*	*-*	*ENTEROCOCCUS FAECIUM*	*-*
84	*-*	*-*	*-*	*-*
85	*-*	*-*	*-*	*-*
86	*ENTEROCOCCUS FAECIUM*	*-*	*-*	*-*
87	*STAPHYLOCOCCUS AUREUS*	*-*	*STAPHYLOCOCCUS AUREUS*	*-*
88	*-*	*-*	*-*	*-*
89	*-*	*-*	*-*	*-*
90	*-*	*ENTEROCOCCUS FAECALIS*	*-*	*-*
91	*-*	*-*	*-*	*-*
92	*-*	*E COLI*	*E COLI*	*-*
93	*-*	*ENTEROCOCCUS FAECIUM*	*-*	*-*
94	*-*	*-*	*-*	*-*
95	*-*	*-*	*-*	*-*
96	*-*	*-*	*E COLI*	*E COLI*

97	-	-	-	-
98	-	-	-	-
99	-	-	-	-
100	-	-	-	-

Supplementary Table 2. Bacteria identifications from bacterial DNA detected in blood of LT recipients before and during the first month after LT (A), and bacteria identifications in liver donors and their recipients (B).

A)

Bacterial DNA IDs before LT		Bacterial DNA IDs after LT
Peripheral blood (n=26)	Portal blood (n=24)	Peripheral blood (n=34)
Escherichia coli (10)	*Escherichia coli (10)*	*Escherichia coli (13)*
klebsiella pneumoniae (5)	*klebsiella pneumoniae (4)*	*klebsiella pneumoniae (4)*
Staphylococcus aureus (4)	*Staphylococcus aureus (3)*	*Morganella morganii (1)*
Enterococcus faecium (2)	*Shigella fleneri (1)*	*Yersinia tuberculosis (1)*
Proteus vulgaris (1)	*Enterococcus faecium (2)*	*Campilobacter jejuni (1)*
Proteus mirabilis (1)	*Streptococcus pneumoniae (2)*	*Citrobacter freundi (1)*
Streptococcus pneumoniae (1)	*Proteus vulgaris (2)*	*Staphylococcus aureus (3)*
Streptococcus agalactiae (1)		*Staphylococcus haemoliticus (1)*
Enterobacter spp. (1)		*Staphylococcus lugdunensis (1)*
		Enterococcus faecium (3)
		Enterococcus faecalis (1)
		Streptococcus pneumoniae (3)
		Enterobacter cloacae (1)

LT: liver transplantation

B)

Donor	Recipient	
	Before LT	After LT
Klebsiella pneumoniae	-	-
Escherichia coli	-	-
Streptococcus pneumoniae	-	*Escherichia coli*
Escherichia coli	*Staphylococcus aureus*	*Streptococcus pneumoniae*
Proteus vulgaris	-	-
Escherichia coli	*Escherichia coli*	*Escherichia coli*
Staphylococcus aureus	-	-
Staphylococcus aureus	-	*Streptococcus pneumoniae*

LT: liver transplantation

Supplementary Table 3. Clinical complications of patients after LT.

Complications during the first 12 months after LT	*n (0-1 month)*	*n (1-12 months)*	*n (total)*
CMV infection	2	26	28
Bacterial infectious complications	14	13	27
Bilary / pancreatic complications	8	10	18
Death	10	6	16
Intraabdominal collection / haemoperitoneum	13	2	15
Graft rejection	6	6	12
Fever of unknown origin	4	6	10
Kidney failure	8	1	9
Ascites after LT	3	3	6
Herpes zoster infection	2	4	6
Tacrolimus neurotoxicity	4	1	5
Evisceration	4	1	5
Diarrhea	1	4	5
Surgical wound seroma	5	-	5
Secondary abdominal wound closure	-	5	5
Upper gastrointestinal bleeding	4	-	4
Atrial fibrillation	3	1	4
Leg cellulitis	2	2	4
Neutropenia	-	3	3
Herpes simplex infection	1	2	3
Herpes esophagitis	3	-	3
Early partial hepatic artery thrombosis	3	-	3
Secondary sclerosing cholangitis	-	3	3
Hypertensive crisis	-	2	2
Pleural effusion	-	2	2
Supraventricular tachycardia	2	-	2
Intraabdominal haematoma	2	-	2
Duodenal ulcer bleeding	2	-	2
Confusional syndrome	2	-	2
Respiratory failure	2	-	2
Viral gastroenteritis	-	2	2
Hepatocarcinoma distant metastasis	-	2	2
Diffuse biliary ductopenia	-	1	1
Urethral stenosis	-	1	1
Lung cancer distant metastasis	-	1	1
Haemophagocytic syndrome	-	1	1
Epigastric pain syndrome	-	1	1
Angiodermatitis	-	1	1
Suppurative chondritis of the rib cartilages	-	1	1
Intestinal ischaemia	-	1	1
Ischemic optic neuropathy	-	1	1
Lower gastrointestinal bleeding	-	1	1
Deep venous thrombosis	-	1	1
Haemorrhagic shock	-	1	1
Left colon adenocarcinoma	-	1	1
Functional abdominal pain	-	1	1
IgA mesangial glomerulonephritis	-	1	1
Gastroenteritis	-	1	1
Neuralgia	-	1	1
Portal thrombosis	-	1	1
Hepatocarcinoma recurrence	-	1	1
Acute diverticulitis	-	1	1
Drug-induced pancytopenia	-	1	1
Peritoneal sarcoidosis	-	1	1
Liposarcoma recurrence	-	1	1
Seizure	1	-	1
Incarcerated umbilical hernia	1	-	1
Duodenal perforation	1	-	1
Leucocytosis	1	-	1
Hyponatremia and acute liver failure	1	-	1
Antral ulcer	1	-	1
Acute gout arthritis	1	-	1
Wound haematoma	1	-	1
Cardiogenic shock	1	-	1
Hepatic artery kinking	1	-	1
Cephalic vein thrombosis	1	-	1
Cardiorespiratory arrest	1	-	1
	**122**	**130**	**252**

Supplementary Table 4. Variables significantly related with developing clinical complications during the first year after LT.

Univariate Analysis

	Patients with complications during the first year after LT	N (%) or (pg/mL)	OR (95%CI)	*P value*
BactDNA at baseline	no	5 / 37 (13.5%)	3.200 (1.158 - 10.409)	0.034
	yes	21 / 63 (33.3%)		
TNF-alpha at baseline	no	22.61 ± 25.34	1.019 (1.005 - 1.037)	0.014
	yes	40.11 ± 36.62		
IL-6 at baseline	no	27.38 ± 29.18	1.015 (1.003 - 1.030)	0.026
	yes	45.60 ± 41.43		
BactDNA during the first 30 days after LT (yes)	no	7 / 37 (18.9%)	3.214 (1.278 - 8.953)	0.017
	yes	27 / 63 (42.8%)		
TNF-alpha at 30 days	no	20.23 ± 28.88	1.017 (0.999 - 1.017)	0.006
	yes	44.42 ± 44.43		

Multivariate Analysis

	OR (95%CI)	*P value*
BactDNA at baseline	1.133 (0.198 - 6.13)	0.882
TNF-alpha at baseline	1.035 (0.962 - 1.119)	0.364
IL-6 at baseline	0.974 (0.914 - 1.034)	0.406
BactDNA during the first 30 days after LT (yes)	0.075 (0.001 - 21.421)	0.388
TNF-alpha at 30 days	1.061 (0.960 - 1.012)	0.264

Supplementary Table 5. Association between the presence of bactDNA during the first month after LT and the inflammatory cytokine levels at 30 days with an increased risk of death, graft rejection and CMV infection during the one-year follow-up.

CMV (univariate analysis)					
Independent Variables	No	Yes	Beta coeff.	95% CI	p
Bacterial DNA No/Yes	50/21	16/13	1.94	0.79 – 7.74	0.15
TNF-alpha	30.9±38.3	46.6±45.6	1.01	0.99 – 1.02	0.08
IL-6	55.7±44.9	69.4±54.9	0.9	0.98 – 1.01	0.47
Bacterial infection (univariate analysis)					
Independent Variables	No	Yes	Beta coeff.	95% CI	p
Bacterial DNA No/Yes	47/26	19/8	0.76	0.29 – 1.98	0.58
TNF-alpha	36.8±42.0	32.0±38.6	1.00	0.99 – 1.01	0.60
IL-6	62.9±49.1	51.9±45.7	1.00	0.99– 1.01	0.32
Biliary/pancreatic complications (univariate analysis)					
Independent Variables	No	Yes	Beta coeff.	95% CI	p
Bacterial DNA No/Yes	55/28	11/5	1.07	0.36 – 3.20	0.90
TNF-alpha	34.1±40.2	38.7±44.6	1.00	0.99 – 1.02	0.48
IL-6	59.3±47.8	57.6±48.8	1.00	0.99 – 1.01	0.80
Exitus (univariate analysis)					
Independent Variables	No	Yes	Beta coeff.	95% CI	p
Bacterial DNA No/Yes	54/30	12/4	0.60	0.16 – 1.90	0.41
TNF-alpha	36.2±40.3	31.4±45.4	1.00	0.98 – 1.01	0.66
IL-6	62.2±49.0	45.1±41.9	0.99	0.98 – 1.00	0.20
Intraabdominal collection (univariate analysis)					
Independent Variables	No	Yes	Beta coeff.	95% CI	p
Bacterial DNA No/Yes	57/28	9/6	1.36	0.44 – 4.19	0.60
TNF-alpha	34.6±41.3	40.3±39.9	1.00	0.99 – 1.02	0.62
IL-6	59.7±46.8	61.0±57.3	1.00	0.99 – 1.01	0.93
Rejection of transplant (univariate analysis)					
Independent Variables	No	Yes	Beta coeff.	95% CI	p
Bacterial DNA No/Yes	59/31	7/3	0.82	0.17 – 3.16	0.78
TNF-alpha	35.9±41.6	31.4±36.2	1.00	0.98 – 1.01	0.74
IL-6	60.6±49.7	49.0±30.0	0.99	0.98 – 1.01	0.47

Supplementary Figure 1. Relationship between microbiologic culture and sequencing analysis identification of bacterial species in patients.

Microbiologic cultures before LT (n=17)	Bacterial DNA IDs before LT (n=26)
Escherichia coli (AF)	***Escherichia coli***
	Klebsiella pneumoniae
Escheria coli; Pseudomonas	***Escherichia coli***
Escherichia coli	***Escherichia coli***
Klebsiella Pneumoniae	***Klebsiella Pneumoniae***
Klebsiella Pneumoniae	***Klebsiella Pneumoniae***
	Staphylococcus aureus
	Escherichia coli
	Escherichia coli
	Enterobacter spp.
	Proteus vulgaris
	Streptococcus agalactiae
	Klebsiella pneumoniae
Escherichia coli	***Escherichia coli***
	Staphylococcus aureus
	Escherichia coli
	Proteus mirabilis
Staphylococcus haemolyticus	*Staphylococcus aureus*
Enterobacter cloacae	*Escherichia coli*
Staphylococcus lugdunensis	*Streptococcus pneumoniae*
Escherichia coli	***Escherichia coli***
Streptococcus parasanguinis	
Enterococcus faecium	***Enterococcus faecium***
	Staphylococcus aureus
Acetinobacter Baumanii; Enterococcus faecalis	
Escherichia coli	***Escherichia coli***
Enterococcus faecium	
	Klebsiella pneumoniae
Enterococcus faecium; Escherichia Coli	***Enterococcus faecium***

AF: ascitic fluid; LT: liver transplantation

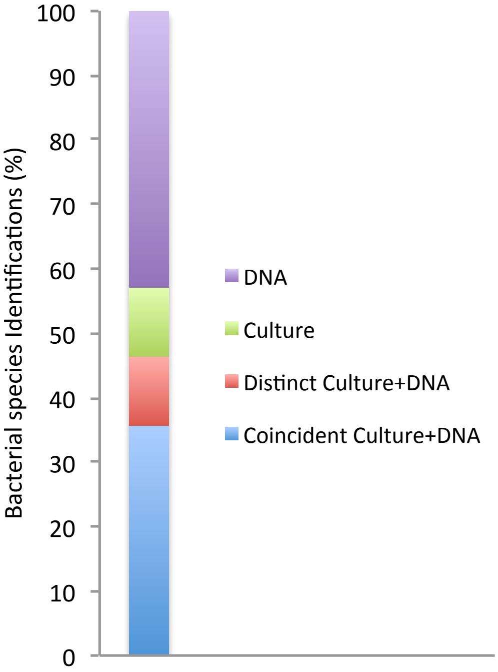

Supplement: Supplementary file 1 — Supplementary Dataset 1 [file 41598_2018_36904_MOESM1_ESM.pdf]
